# Introduction to Photocatalytic Materials for Clean Energy, Renewable Chemicals production, and Sustainable Catalysis

**DOI:** 10.1039/d4na90075h

**Published:** 2024-07-08

**Authors:** Rajeev Ahuja, Rajendra Srivastava

**Affiliations:** a Department of Physics and Astronomy, Condensed Matter Theory, Materials Theory Division, Uppsala University Uppsala 75120 Sweden rajeev.ahuja@physics.uu.se; b Department of Physics, Indian Institute of Technology Ropar Rupnagar Punjab 140001 India; c Department of Chemistry, Catalysis Research Laboratory, Indian Institute of Technology Ropar Rupnagar Punjab 140001 India rajendra@iitrpr.ac.in

## Abstract

Professor Rajeev Ahuja & Professor Rajendra Srivastava introduce the *Nanoscale Advances* themed collection on Photocatalytic Materials for Clean Energy, Renewable Chemicals production, and Sustainable Catalysis.
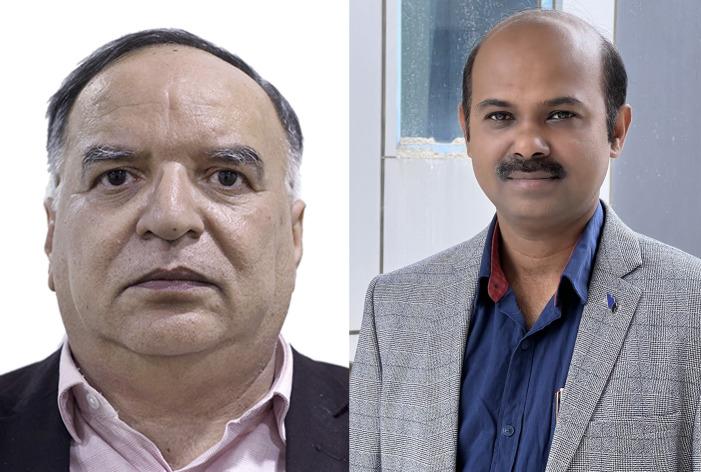

The growing population has increased the demand for energy sources and commodity chemicals, which are mostly fulfilled by fossil-fuel sources. The excessive use of fossil fuels pollutes the air and water. The atmospheric CO_2_ concentration reached 417.5 ppm in 2022, which is ∼50% higher than the CO_2_ concentration during the pre-industrial era (mid-1700).^1^ The last 40 years have produced approximately 50% of the cumulative CO_2_ emitted since 1750, a direct consequence of the growing population and the increasing fossil-fuel consumption for producing energy and chemicals.^2^ According to the Paris Climate Agreement, to limit the global temperature rise to 1.5 °C above that of the pre-industrial era and to avoid temperature-driven catastrophic climatic events, CO_2_ emissions must drop to zero by 2050.^3^ Lowering of the atmospheric CO_2_ concentration can be accomplished by adopting three main strategies, namely: (a) reducing atmospheric CO_2_ emissions *via* CO_2_ capture & storage, and converting CO_2_ into value-added chemicals;^4^ (b) renewable chemicals and fuel production employing sustainable energy sources; and (c) environmental remediation employing sustainable energy sources.

Sunlight provides a thousand times more energy to the earth's surface each year than global energy consumption annually, offering a sustainable energy source.^5^ Harnessing the power of sunlight, photocatalytic materials need to be developed to catalyze chemical reactions that hold promise for clean energy production, synthesizing renewable chemicals, and mitigating environmental pollutants. The early research on solar-light-driven technologies, namely photocatalysis (PC) and photoelectrocatalysis (PEC), mainly focused on H_2_ fuel generation *via* H_2_O splitting and producing valuable organic compounds, such as pharmaceutical building blocks and fine chemicals. However, the recent focus has shifted towards producing hydrocarbon fuels and chemicals from CO_2_, intending to decarbonize the earth's atmosphere.^5^ The photocatalytic conversion of CO_2_ into methane, methanol, formaldehyde, acetic acid, and higher hydrocarbon fuels and alcohols is attracting growing interest.^6–8^ The recent focus has shifted towards producing renewable chemicals, fuels and NH_3_, and removing water and air pollutants employing light energy, as it offers an alternative strategy to produce value-added chemicals and hydrocarbon fuels currently obtained from non-renewable fossil fuels.

Air pollution remains a pressing global issue due to the continuous release of NO_*x*_ from fossil-fuel combustion, contributing to photochemical smog and acid rain, and adversely affecting human health and ecosystems. Semiconductor photocatalysis offers a sustainable solution by harnessing solar energy to convert NO_*x*_ into less-harmful compounds under mild conditions;^9,10^ efficient photocatalysts enhance NO removal and selectively convert NO to environmentally benign products like NO_3_^−^, minimizing secondary emissions. Developing advanced photocatalytic systems tailored for effective NO_*x*_ reduction holds promise for mitigating air pollution and advancing environmental sustainability. In addressing water pollution caused by industrial dyes, photocatalytic dye degradation plays a crucial role.^11,12^ When irradiated with light, the photocatalyst generates reactive oxygen species (ROS) such as hydroxyl radicals (˙OH) and superoxide anions (O_2_˙^−^). These highly reactive ROS oxidize dye molecules, breaking them down into less-harmful compounds like water and carbon dioxide.

Photoelectrochemical water splitting and the photocatalytic hydrogen evolution reaction (HER) are two advanced approaches for sustainable H_2_ production from water using sunlight.^13^ PEC utilizes semiconducting materials to generate H_2_ and O_2_ through efficient optimization of material nanostructures, heterojunction engineering, and surface functionalities. The HER, on the other hand, focuses specifically on using semiconductor photocatalysts to drive the reduction of protons (H^+^) from water to produce hydrogen gas (H_2_). This process harnesses the absorption of sunlight by photocatalysts to generate electron–hole pairs. PEC water splitting and photocatalytic HER support the transition to a hydrogen-based economy by offering a renewable, environmentally friendly alternative to fossil fuels, thus advancing global sustainability efforts.

All of the above-discussed photocatalytic applications for environmental remediation can be realized by synthesizing efficient photocatalytic materials. This can be accomplished by adopting modern synthetic strategies and integrating advanced materials science with photocatalytic principles to unlock unprecedented opportunities to harness solar energy. Innovations in catalyst design, such as creating multi-component systems and incorporating plasmonic materials, have shown significant promise in enhancing photocatalytic performance. One of the primary strategies in photocatalytic synthesis is the development of semiconductor-based materials with tailored nanostructures. By engineering the size, shape, and composition of semiconductor nanoparticles, researchers can significantly improve light absorption, charge separation, and surface reactivity. Modifications such as doping with metal or non-metal elements, creating heterojunctions, and surface functionalization improve photocatalytic performance by extending the light absorption range into the visible spectrum and enhancing charge separation.

This collection of reviews, minireviews and research articles highlights the pivotal role of multifunctional nanomaterials in advancing the efficiency, selectivity, and scalability of photocatalytic processes in harnessing clean energy, producing renewable chemicals, and developing sustainable catalytic routes.

The collection begins with a comprehensive minireview by Wang *et al.* (https://doi.org/10.1039/D3NA00837A), on metal–organic framework (MOF)-based photocatalytic materials. MOFs have high crystallinity, large surface area, and variable metal nodes, linkers, and functional ability, which makes them ideally suited for the design and development of photocatalytic processes. The minireview elucidates the design rules and principles for engineering MOFs in manipulating the interfacial charge dynamics in MOFs for enhanced photocatalytic applications.

Harnessing graphene-based nanocomposites for photocatalytic applications leverages graphene's exceptional electronic conductivity and large surface area, enhancing charge transfer and overall photocatalytic efficiency. Integrating graphene with semiconductor materials promotes efficient light absorption and charge separation, making it a promising approach for advanced photocatalytic systems. Potbhare *et al.* (https://doi.org/10.1039/D3NA01071F) contributed an updated review on graphene-based metal oxide nanocomposites, emphasizing their dual functionality in photocatalysis and energy storage systems. This review summarises recent advancements in materials synthesis techniques and highlights their potential impact on sustainable catalytic processes and environmental remediation strategies.

Innovative functionalization strategies enhance photocatalysts' efficiency and selectivity and demonstrate their applicability in selective chemical transformations and environmental pollutant remediation. Vega-Fernández *et al.* (https://doi.org/10.1039/D4NA00149D) introduce novel strategies for photocatalytically functionalizing thin-layer membranes using a monomer truncation strategy, showcasing organic polymer films with enhanced photocatalytic activity. This photoactive film demonstrated effective luminescence properties, enabling the oxidation of sulfides to sulfoxides and the reduction of aryl bromines.

Enhancing photocatalytic efficiency for NO_*x*_ removal focuses on refining materials properties and integrating advanced nanostructures, significantly boosting the degradation rates of nitrogen oxides. These advancements offer more efficient and environmentally friendly solutions for air pollution control. Pham *et al.* (https://doi.org/10.1039/D4NA00035H) explore the efficient photocatalytic removal of NO_*x*_ pollutants using triangular Ag nanoparticles coupled with TiO_2_. This study underscores significant enhancements in photocatalytic efficiency while minimizing toxic byproducts, highlighting the role of plasmonic nanomaterials in sustainable air purification technologies.

Utilizing semiconductor photoelectrode materials for solar hydrogen production is a promising approach to generate clean, green energy with a zero-carbon footprint while minimally altering existing infrastructure. Sitaaraman *et al.* (https://doi.org/10.1039/D4NA00088A) present an innovative nanostructured tandem cell for unassisted solar water splitting, featuring FeOOH/NiOOH-coated BiVO_4_ as a photoanode and TiO_2_-protected Cu_2_O/CuO as a photocathode. This work showcases efficient hydrogen evolution under sunlight, offering a sustainable pathway toward renewable hydrogen production.

Combining semiconductors with complementary electronic properties to form heterojunctions optimizes charge transfer and separation, enhancing photocatalytic activity through reduced recombination losses. Luo *et al.* (https://doi.org/10.1039/D3NA01091K) introduce an S-scheme heterojunction of BiVO_4_/VS-MoS_2_ for efficient photocatalytic nitrogen fixation, which is very attractive for the sustainable production of NH_3_. This study highlights significant advancements in utilizing nanoscale heterostructures to overcome kinetic barriers and achieve high-efficiency nitrogen reduction under ambient conditions, which is crucial for sustainable agriculture and chemical synthesis.

Present methods of nanomaterials synthesis often struggle to achieve precise control over synthetic outcomes, primarily due to poorly defined reaction protocols, and this challenge becomes multi-fold when agricultural waste is used as a direct source. Verma *et al.* (https://doi.org/10.1039/D3NA00596H) explore the viability of utilizing lignocellulosic waste, specifically sugarcane press mud, for synthesizing ZnO nanoparticles using three distinct precursor salts. These nanoparticles were subsequently evaluated for their effectiveness in the photocatalytic degradation of rhodamine dyes.

Surface plasmon resonance (SPR)-based heterogeneous photocatalysts have demonstrated significantly enhanced photocatalytic efficiency under visible light. The effectiveness of plasmonic photocatalysis depends on various factors within the system, including the choice of specific metals or noble metals and the supporting materials utilized. Rani *et al.* (https://doi.org/10.1039/D3NA00583F) investigate microwave-assisted synthesis of Ni–NiO@Ni_2_CO_3_(OH)_2_ core–shell@sheet hybrid nanostructures for plasmonic photocatalysis. Their work demonstrates enhanced hydrogen evolution through flexible nanosheets, highlighting the potential of cheaper metals in solar-driven water-splitting technologies.

In conclusion, this themed collection encapsulates the forefront of research and development in photocatalytic materials, offering insights into novel materials design, synthesis methodologies, and their diverse applications in clean energy and environmental (air and water) remediation. As guest editors, we sincerely thank the authors for their pioneering contributions and the editorial team of Nanoscale Advances for their support in curating this comprehensive exploration of photocatalytic innovations. This themed collection will catalyze interdisciplinary collaboration, fostering new ideas and practical innovations in pursuing sustainable energy solutions.
